# “French Phage Network” Annual Conference—Fifth Meeting Report

**DOI:** 10.3390/v12040446

**Published:** 2020-04-14

**Authors:** Floriane Laumay, Amel Chaïb, Romain Linares, Cécile Breyton

**Affiliations:** 1 Genomic Research Laboratory, Geneva University Hospitals, CH-1211 Geneva, Switzerland; floriane.laumay@genomic.ch; 2 ISVV, EA4577 Œnologie, University of Bordeaux, Villenave d’Ornon, 33140 Bordeaux, France; amel.chaib@u-bordeaux.fr; 3 CNRS, CEA, IBS, University Grenoble Alpes, F-38000 Grenoble, France; romain.linares@ibs.fr

**Keywords:** bacteriophages, Ecology and Evolution, Phage Therapy and Biotechnology, Phage–Structure and Assembly, Host Interaction

## Abstract

Attracting about 100 participants, the fifth edition of our French Phages.fr annual conference was once more a success. This year’s conference took place at the Institute for Structural Biology on the European Electron and Photon Campus in Grenoble, 8–9 October 2019. Similar to previous years, our meeting gathered scientists mainly working in France, from academic labs and hospitals as well as from industry. We also had the pleasure of welcoming attendees from different European countries and even beyond. The conference was divided into four sessions: Ecology and Evolution, Phage Therapy and Biotechnology, Structure and Assembly and Phage–Host Interaction, each opened by a keynote lecture. The talks, selected from abstracts, gave the opportunity for young scientists (especially students and post-docs) to present their project and results in a friendly atmosphere. Poster sessions also favoured interactions and discussions between young researchers and more senior scientists. Here, we provide a summary of the topics developed during the conference.

## 1. Introduction

Bacteriophages or phages, viruses that infect bacteria, are ubiquitous on Earth. Discovered just about 100 years ago, their potential to cure infectious diseases, later called phage therapy, was soon applied [1,2]. During the first half of the 20th century the study of phage by physicists interested in understanding the basis of life, lead to the birth of a new scientific field, Molecular Biology (for a review [3]). During the second half of the 20th century however, phage research declined, due to the increasing number of antibiotics and because many scientists shifted their interest toward more “complex” living systems. Nowadays, with the alarming increase of animal and human pathogen resistance to antibiotics [4], a renewed interest for phage therapy is observed. Together with the rising awareness of the importance of phages in all ecosystems (e.g. [5,6,7]), the potentialities of phages in biotechnology (e.g. [8,9,10]), the progress in structural biology (e.g. [11,12]), and the fascinating complexity of phage ecology and evolution (e.g. [13,14]), the study of phages is back to the frontlines in a wide variety of scientific fields.

**Figure 1:**
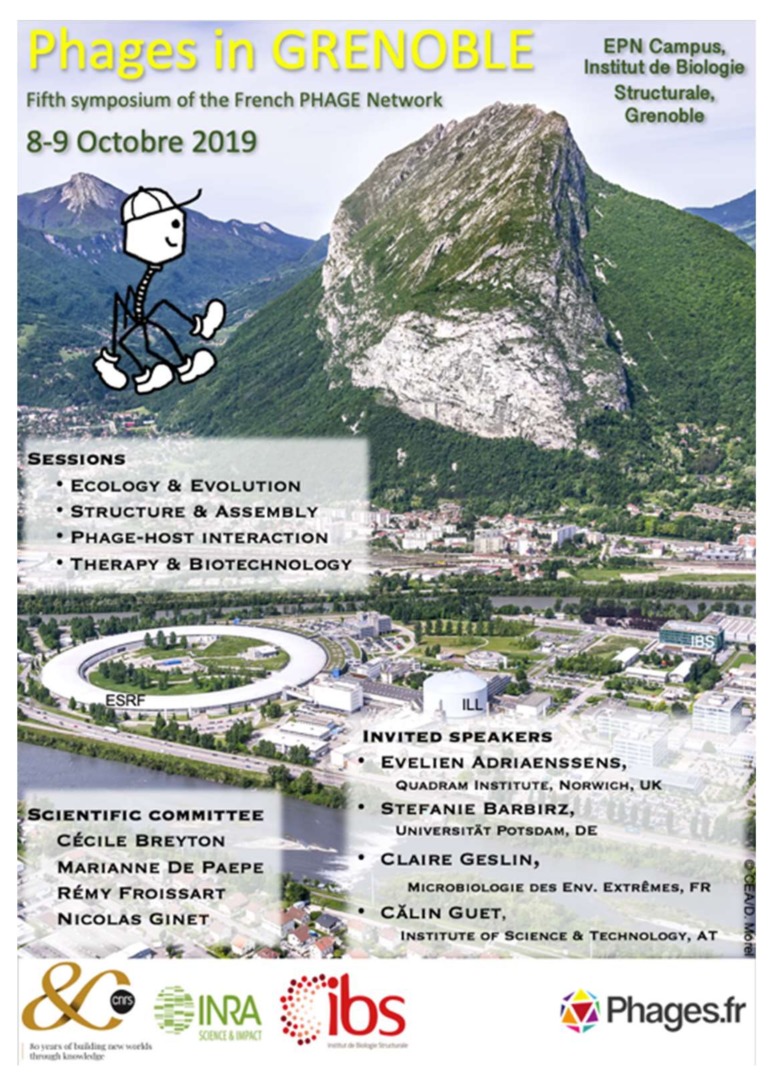
Poster of the Phage in Grenoble Conference.

The Phages.fr network aims at federating researchers mainly located in France who use bacteriophages or their applications in their research, in a very large range of projects (ecology and evolution, molecular mechanisms of infection, genetic regulation, industrial and therapeutic applications, etc.). Our yearly meeting gives them the opportunity to interact, discuss, share experiences and initiate new collaborations. This year, this community met at the Institute for Structural Biology, on the European Photon and Neutron Campus in Grenoble, 8–9 October 2019 (Figure 1). Attracting a total of 98 attendees coming from all over France, but also from Germany, Italy, Belgium, Switzerland, United Kingdom, Austria, Spain, Czech Republic, Hungary, Turkey and Canada, the international exposition of this annual meeting has expanded. However, we deeply regret that a Turkish colleague, coming from Italy, was refused crossing of the French border, thus, she could not attend the meeting and present her topic. The four sessions (21 talks that were selected from abstracts), all chaired by students or post-docs, were opened by a keynote lecture given by guests invited from Austria, France, Germany and the United Kingdom. Discussions were further extended during two speaking sessions (Table 1), lunches, dinners and coffee breaks. Half the attendees were either students (29) or post-docs (15), and gender parity was reached at the level of the attendees, with a majority of women speakers (16 versus 9 men). Two private companies, Myriade and Cellexus, presented their technologies related to a rapid method for counting viral particles and a disposable system for the production of phages, respectively.

## 2. Summary of the Scientific Sessions

### 2.1. Ecology and Evolution

**Evelien Adriaenssens** (Quadram Institute, Norwich, UK) opened this session, chaired by Damien Piel (PhD student, LM2E, Roscoff, France) and Jack Dorling (PhD student, I2BC, Gif–sur–Yvette, France), with a keynote lecture entitled “Phage Taxonomy: what, how and why?”. Phage taxonomy recently experienced an important evolution linked to sequencing technologies and metagenomics. Although tailed phages were historically classified into families based on morphological analysis of their tail structure (Myoviridae, Siphoviridae and Podoviridae), for example, thousands of bacteriophage sequences recently deposited in public databases show little to no sequence homology between members of the same family, challenging the current classification scheme. Being chair of the ICTV (International Committee on Taxonomy of Viruses) Bacterial and Archaeal Viruses Subcommittee, Evelien described how the subcommittee is currently reorganizing the morphology-based families in favour of genome-based ones, using an ensemble of genomic, proteomic and phylogenetic methods. Using the case study of a group of large Myoviruses, the Spounaviruses, now grouped into a new family called Herelleviridae, she presented a large-scale, genome-based taxonomic classification that can robustly be carried into the future, fit for high-throughput and metagenomic analyses. She also discussed the rules for naming newly discovered phage (sub)families and suggested working with taxonomists upon these matters. Finally, she presented a series of useful tools for taxonomy (ViPTree server, GRAVity, vConTACT, VICTOR).

Bacteriophages, as an important group of predators of bacteria in the ocean, are thought to play an important role in controlling fine-scale blooms within these communities. Using the case of the oyster pathogen *Vibrio crassostreae*, **Frédérique Le Roux** (Ifremer, Marine station of Roscoff, France) wanted to understand whether phages are major drivers of microbial abundance and diversity, and how they evolve in response to *Vibrio* resistance. Her team performed seawater and oyster tissue time series sampling over several months and at two distant sites (Brest and Sylt) to assess the dynamic of *Vibrio* and their phages. They showed that their abundances are inversely correlated. Investigating the host range of Vibriophages also strongly suggested local adaptation or co-evolution, the phage populations exhibiting a good connection with *Vibrio* clones. This work also allowed the characterisation of broad host phages to be used for the rational design of phage cocktails against oyster pathogens.

Phages and plasmids are two major groups of mobile genetic elements (MGEs) shaping bacterial evolution. Although literature distinguishes them clearly, several MGEs were reported to be plasmids encoding *bona fide* phage genes (or *vice versa*), enterobacteriophages P1 and N15 being the most prominent examples of the so-called “phage–plasmids”. **Eugen Pfeifer** (research fellow, Institut Pasteur, Paris, France) presented work aiming at better understanding those MGEs through the study of known phage–plasmids, the identification of novel ones and the evaluation of their impact on bacterial evolution. The screening of phage and plasmid databases for typical phage characteristics in plasmids and plasmid features in phages resulted in the prediction of around 500 putative phage-plasmids. Genome-based clustering allowed the determination of 29 phage–plasmid families, each containing two to fifty members, amongst which a few encode many virulence and resistance traits. Future work will aim at understanding the evolution of these phage–plasmids.

Lactic acid bacteria (LAB) as *Streptococcus thermophilus* are widely used for industrial food fermentation, but also generate great interest for their food-grade expression capabilities (proteins or metabolites) [15,16]. To do so, replicating plasmids are often used to introduce genetic material into LAB cells [17,18], but these plasmids need to be maintained into the cells. This can be achieved by several methods, including recently the use of CRISPR–Cas technology [19,20]. **Cécile Philippe** (postdoctoral fellow, Laval University, Quebec City, Canada) demonstrated that streptococcal phage proteins also can be used to increase plasmid stability in LAB cultures and, thus, could be a new tool for food-grade vector applications. Moreover, she showed that this plasmid stability phenotype was not strain-dependant, using various industrial strains.

**Marie–Agnès Petit** (MICALIS, Jouy–en–Josas, France) and her team previously showed that antibiotic resistance genes (ARG) were infrequent in phage genome and virome contigs [21]. Conversely, recent studies suggested that pig fecal samples could be rich in viral ARG, although these conclusions could be impaired due to bacterial DNA contamination [22,23]. Fourteen pig (five piglets and nine adults) fecal samples were processed to obtain clean and pure pig viromes. Subsequent sequencing led to the conclusion that phage contigs reconstructed from these pig viromes were indeed poor in ARG, although some were found on phage contigs from four of the piglet samples. Also, Inoviridae appeared to be much more prevalent in viromes than previously thought [24], especially for pig fecal samples.

The *Ralstonia solanacearum* species complex causes one of the most important plant diseases in the world, bacterial wilt. **Clara Torres–Barceló** (INRA–PACA, Monfavet, France) presented a study aiming at isolating and characterising new *Ralstonia* spp. phages to be used as biocontrol tools. The work also addressed evolutionary questions on the host range of phages, regarding a potential trade-off between the generality and efficiency of phages at controlling their hosts. Forty-two phages were isolated from agricultural samples in Mauritius and the Reunion islands and their host range was tested on 64 *Ralstonia* spp. strains, from local or international origins. Although phages preferentially attacked bacterial strains present in the islands, 18 of them were able to multiply in bacteria never detected in the islands. Phages also exhibited variations in their efficacy at decreasing bacterial growth. Finally, Reunion phages appeared to be quite specialised and efficient against genetically homogeneous local *Ralstonia* spp. strains, whereas Mauritius ones were more generalised but not as competent at controlling all of them. The results of this study suggest that the host range should not be the only selection criteria for phage therapy and that they should integrate the phylogenetic information of the targeted bacteria.

To enable a phage epidemic to occur, phages need to bypass the host immunity, especially the widespread prokaryotic adaptive immune system CRISPR–Cas. **Hélène Chabas** (research fellow, ETH Zürich, Switzerland) investigated why, in some cases, phage infection results in large epidemics or in other cases in phage extinction. Using a stochastic epidemiological model describing CRISPR–phage interactions, she determined that, in the absence of phage evolution, higher probabilities of CRISPR-resistance acquisition increase the probability of phage extinction. However, when phage can escape CRISPR through single mutations, the probability of phage extinction is bimodal: below a certain epidemiological tipping point, phages always remain in the population, whereas above this point, phages are always led to extinction. She then explored whether phages can increase their probability of survival by increasing their mutation probability and found that this has only a minimal effect.

### 2.2. Phage Therapy and Biotechnology Session

The high specificity of phage–host interactions has rapidly led to applications in the agricultural and food industry, as well as in the human clinics. This session was dedicated to the use of phages for human health and activities, and was chaired by **Fernando Clavijo** (PhD student, Laboratory of Bacterial Chemistry, Marseille, France).

It opened with a keynote lecture by **Claire Geslin** (LM2E, Brest (UBO), France). Phages are a driving force in many evolutionary processes, even in extreme conditions such as deep-sea hydrothermal vents. Only a few viruses have been isolated from microorganisms living in these environments. Geslin’s group has characterised some of them. PAV1 and TPV1 are two lemon-shaped viruses from the Thermococcales order that infect hyperthermophilic anaerobic archaea; they have two homologous genes encoding proteins containing a concanavalin A-like lectin/glucanase domain, probably involved in virus–host recognition. MFV1 is the first head–tailed virus isolated from a deep-sea hyperthermophilic archaea. To contrast, MPV1 infects a thermophilic, anaerobic and piezophilic bacterium, and is able to package a 13.3 kb plasmid originating from its bacterial host (lateral gene transfer or molecular piracy events). Overall, Claire provided evidences of a morphological and genetic diversity among viruses of (hyper)thermophilic archaea and bacteria, with a prevalence of lysogeny that might contribute to viral DNA stability under extreme conditions [25].

**David Olivenza** (PhD student, Departamento de Genética, Sevilla, Spain) talked about the development of biosensors for phage detection and phage receptor discrimination. The rapid detection of phages may have important implications in many fields, such as assessing the presence of phages in industrial processes and following up the evolution of phage population during and after therapeutic treatment; however, only a few methods are available to detect specific phages in a given sample. Olivenza’s work consisted of using opvAB-*gfp*-based construction as a biosensor for detecting phages using the O-antigen as a receptor. *Salmonella enterica* opvAB operon allows the production of short chained-LPS and provides resistance to phages recognizing LPS as a receptor. Upon phage infection, cells expressing opvAB operon are the ones to be selected. The fusion of the operon with a *gfp* reporter gene allowed the high sensitivity monitoring of LPS-binding phages in various media.

The session continued with a presentation of **Ildikó Nagy** (Enviroinvest Environmental and Biotechnological Corporation, Hungary). The bacterial leaf blight is responsible for important economic losses in rice production, especially in Asia and in western Africa. The challenge is to find pesticides against the causative agent, *Xanthomonas oryzae* pv. *oryzae* (*Xoo*), which are both effective and safe for the environment. Nagy’s laboratory characterised twelve *Xoo* bacteriophages from Asia (from water or soil in the Philippines and Vietnam) and from France (from a laboratory in Montpellier). Genome-based comparisons allowed the establishment of the phylogenetic relationship among different phages, with the identification of a 322 bp conserved intergenic region. Additionally, all phages were efficient against *Xoo* from Asia, but not against *Xoo* from Africa and *X. oryzae* pv. *citri* strains.

Beyond the presence of multi-resistant bacteria, infections can be complicated to eradicate due to their location, the presence of prosthetic devices or the immunological context. **Alexandre Bleibtreu** (Hôpital Pitié Salpêtrière, Paris, France) described the first utilisation of phages combined with antibiotics for treating a patient suffering from an extradural empyema. The patient was a 29 year-old woman with a type I neurofibromatosis and a pilocytic astrocytoma (in 2007), further complicated by an extradural empyema. Despite the use of appropriate antibiotics and surgeries, the infection due to methicillin-sensitive *Staphylococcus aureus* persisted. Clinical and microbiological failure status was declared at the end of 2018. At the request of the patient and her family, she was allowed to receive a personalised cocktail of two phages produced by Pherecydes Pharma, in combination with intravenous injection of dalbavancin. The infection was successfully cured; she has left the hospital a few months after phage administration.

This session ended with a lecture by **Raphaëlle Delattre** (research fellow, Pasteur Institute, Paris, France) presenting a summary of the diverse topics discussed this year by the Comité Scientifique Spécialisé Temporaire de l’Agence Nationale de Sécurité du Médicament et des Produits de Santé about phage therapy. Regarding the compassionate administrations of bacteriophages in France, the ten patients treated were in complex clinical situations, sometimes without an alternative. The majority of them suffered from joint and bone infections due to *Pseudomonas aeruginosa* or *Staphylococcus aureus*. The specificities of phage therapy, the difficulty to access phages, the lack of clinical trials (activity and fate of phages, modes of administration) and the misinformation about phage therapy were evoked. The committee was favourable to the creation of a national platform managing patients’ requests and a phage library, and to the promotion of academic phage production.

### 2.3. Structure and Assembly Session

This small session, chaired by **Müge Senarisoy** (research fellow, I2BC, Orsay, France), was introduced by the keynote lecture of **Stefanie Barbirz** (University of Potsdam, Germany), who dissected the biophysics of the interactions between the different families of phages (short-, long flexible- and long contractile-tailed phages) and the outer surface of Gram-negative bacteria, covered by lipopolysaccharide (LPS). Very elegant experiments were performed on either purified LPS preparations or LPS-containing outer-membrane vesicles to explore the effect of LPS composition and environment on the kinetics of binding and of DNA ejection of the different families of phages. Stefanie could show that tail morphology dictated DNA ejection velocity, short tailed phages being much slower than long tailed phages, and that binding and cell wall perforation can be dissociated in short-tailed phages, depending on temperature, whereas it is coupled for the other phages [26,27].

The two following talks were given by researchers from the Institute for Structural Biology, Grenoble, studying the structure of the coli-siphophage T5. **Romain Linares** (research fellow), presented his latest data on the structural analysis of the tail of phage T5. Taken from cryo grids of purified T5 tails obtained from a capsid Amber mutant [28] and data collected on the high-end Krios electron microscope of the ESRF (European Synchrotron Radiation Facility), Romain could calculate a map of the tip (end of the tail tube) of the tail of phage T5 at 3.5 Å resolution (see also [29]). Structural analysis shows remarkable structure conservation between the different proteins, but also with proteins of homologous functions in tails of myophages, a type 6 Secretion System, and other phage-derived perforating apparatus.

During the vulnerable time during which the bacterium is turned into a viral factory, phages protect their host from over-infection. Regarding T5, this mechanism is mediated by a periplasmic lipoprotein, Llp, targeted to the inner leaflet of the outer membrane, which binds the phage receptor FhuA. Llp’s main biological function probably also is to prevent the inactivation of progeny phage by active receptors present in the outer-membrane debris of lysed cells and increasing their chance of infecting a new host. **Séraphine Degroux** (PhD student) presented her project aiming at deciphering this immunity mechanism using a structural approach. She already produced the Llp protein as an acylated form and as a soluble form. The proteins behaved properly, auguring well for future studies.

### 2.4. Phage–Host Interaction Session

This large session, chaired first by **Maud Billaud** (PhD student, MICALIS, Jouy–en–Josas, France) then by **Adelaïde Renard** (PhD student, Bactéries et risques materno-foetal, Tours, France), started with a keynote lecture by **Călin Guet** (Institute of Science and Technology, Austria) entitled “The cost of immigration control and the benefits of illegal Immigration”, a metaphoric way to describe the impact of Restriction–Modification (RM) systems on prophage acquisition. This study lies on the assumption that RMs could be costly for their hosts. Conversely, it was noticed that the more bacterial genomes contain RMs, the more they contain prophages. It, therefore, was assessed whether RM efficacy could differ during lysogenic and lytic infections. Using *E. coli* and a Lambda phage, it was demonstrated that carrying RMs is costly for the host at the individual level, which relies on self-restriction phenomena. Efficiency of plating and lysogenisation measurements showed that RM activities do not differ within lytic and lysogenic infections, but that, at the population level, RMs promote prophage acquisition. An explanation would be that RMs delay population infection by the phage until bacteria reach high densities, where the lysogenic cycle is predominant [30]. Immigration control, therefore, is used for illegal immigration purposes that benefit the host by conferring upon it new traits.

The second talk of the session was presented by **Marie Vasse** (research fellow, ETH, Switzerland), where *Myxococcus xanthus* was used to study the impact of phages on its host lifecycle. *M. xanthus* is a bacterium that displays a complex lifecycle alternating between vegetative cells and fruiting bodies that form upon starvation, leading to the production of spores [31]. When bacteria are challenged with the MX1 virulent phage, germination is delayed, indicating that spores might sense phage or infected/dead cell components. Moreover, the fruiting bodies aspect can be modified when exposed to different phages.

The session continued with a presentation by **Pauline Misson** (PhD student, MICALIS, Jouy–en–Josas, France) about the impact of *E. coli* LF82 prophages on the macrophage-induced persistence of their host. *E. coli* LF82 is frequently associated with the microbiota of Crohn’s disease patients. This strain can survive within macrophages and produce persisters (i.e. phenotypic variants with low metabolic activity and transient resistance to antibiotics) [32]. LF82 harbours five prophages. One of them, Gally, a P22-homologue, is most frequently found in *E. coli* associated with Crohn’s disease [33] and seems to have a positive impact on the macrophage-induced persistence of its host *E. coli* LF82. Three Gally genes were shown to be overexpressed within macrophages, and their inactivation led to a 3,4-fold less persister production. These genes could be implicated in a non-canonical DNA recombination mechanism, combining homologous and non-homologous recombination to promote DNA repair in persister cells.

**Amel Chaïb** (PhD student, ISVV Œnologie, University of Bordeaux, France) made a presentation on lysogeny in *Oenococcus oeni*, the main agent responsible for malolactic fermentation of wine. Although prophage prevalence in *O. oeni* species is high, its roles on bacterial fitness are still poorly understood. An elegant, colorimetric method, to discriminate lysogenic derivatives of *O. oeni* was presented (lysogens grow red on red grape juice agar medium, while non-lysogenic clones are white). It simplified the construction of isogenic pairs of strains that do or do not carry prophages for further characterization. Different approaches demonstrated that a white to red colony colour switch is linked to prophage spontaneous induction within the intracolonial population. Red grape polyphenolic compounds were shown to interact with dead cell material. The method was further adapted to other LAB species [34].

**Mehdi El Sadek Fadel** (PhD student, I2BC, Gif–sur–Yvette, France) then reported a study on the molecular mechanism of selective viral DNA recognition and packaging of bacteriophage SPP1. Encapsidation depends on the recognition of a *pac* sequence present on the SPP1 genome by the viral terminase [35]. Despite the specificity of the terminase for a *pac* sequence, bacterial DNA encapsidation can occur and lead to generalised transduction. Interactions between SPP1 terminase and *pac* were characterized through sequential deletions and substitution mutagenesis of the *pac* region. Results show that a large part of a *pac* sequence is dispensable. Extensive degenerations of *pacL* and *pacR* sub-regions are compensated through suppressor mutations leading to overexpression of the terminase complex or changes in the gp1 protein, respectively, indicating the importance of the concerned sites for viral DNA recognition and cleavage.

**Luis Ramirez** (PhD student, I2BC, Orsay, France) presented his work on the *E. coli* T5 phage early gene function in host takeover. The T5 phage delivers its DNA in two sequential steps into the bacterial cell. First, only 8% of the T5 genome is injected, allowing the expression of 17 early genes, then injection resumes. Products of these early genes cause host genome degradation and deactivate its defence mechanisms. Among them, only A1 and A2, a putative DNase and a putative transcriptional regulator, respectively, are known to be essential [36]. As a first step in studying the significance of early genes in T5 infection, genetic engineering strategies were successfully developed to introduce amber mutations, as well as single and multiple gene deletions in T5. Several T5 mutants exhibited changes during the latent period, burst size and host lysis. These data help to identify critical T5 early genes needed for host takeover.

**Naoual Derdouri** (PhD student, Laboratoire de Chimie Bactérienne, Marseille, France) then presented her work on the AppY protein, encoded by an *E. coli* K-12 DLP12 defective prophage. AppY is a transcriptional regulator of the AraC family, also suspected to improve RNA polymerase sigma S factor (RpoS) stability under stress conditions [37]. During the exponential growth phase, RpoS interaction with RssB triggers its degradation by the ClpXP protease. When needed, the RpoS degradation pathway is inhibited by Ira proteins that titrate RssB. Mutation within the HTH motif of the AppY regulator indicated that its transcriptional regulator function is not needed for RpoS stabilisation. Different molecular approaches demonstrated that AppY interacts directly with RssB and that the full-length protein is required, its N-terminal conformation is particularly important. AppY might be a new member of the Ira proteins family. This work shows, for the first time, a prophage-encoded protein-stabilising RpoS.

**Audrey Labarde** (engineer, I2BC, Gif–sur–Yvette, France) presented her work about the sequential program of phage SPP1 genome replication during infection of *Bacillus subtilis*. She found that viral replication occurs in a specific focus in the cytoplasm [38] and that large quantities of SPP1 DNA are synthesised within the first thirty minutes following infection. This involves the rapid and massive recruitment of the bacterial replisome machinery at this focus. This process is orchestrated by the SPP1 helicase gp40, known to bind the DnaG primase as well as DnaX, a subunit of the DNA polymerase III [39,40]. Audrey showed that these three partners are found in the viral DNA focus a few minutes after infection and that their concentration increases over time. She finally observed that the viral DNA compartment is dynamic and takes different shapes during the infection process.

**Quentin Lamy–Besnier** (PhD student, Pasteur Institute, Paris, France) presented an overview of the utilisation of the Viral Host Range database (VHRdb) developed by his team. The identification of host–virus pairs can be difficult and time-consuming due to the accumulation of information and the variety of appellations in public databases. This lack of uniformity results in publication of host ranges that are often incomplete. Additionally, the results of host range experiences performed routinely in many laboratories are rarely publicly available. The ambition of the VHRdb is to provide a tool for the analysis of the host range of viruses and to become a public resource to centralise the experimental information linking viruses to hosts. The VHRdb will be launched during the first semester of 2020.

The session ended with the presentation by **Kovacs Tamas** (Enviroinvest Environmental and Biotechnological Corporation, Hungary), who proposes a COST Action on bacteriophage research and applications. COST (European Cooperation in Science and Technology) is a funding organisation for research and innovation networks. It aims at financing conferences, workshops, training and short missions between members of the network. Thirty-nine countries are eligible, and at least seven should be included in a COST. COST actions are bottom-up networks, and the new COST Kovacs proposes to setup would include five working groups: Ecology, Evolution and Taxonomy; Phage–Host interaction; Structure (including genome); Applications in Plant Protection; Application in Human and Animal Health. To register, please follow the www.europhage.eu link.

## 3. Conclusions

Similar to previous years, our French Phage Network annual conference favoured many discussions and exchanges in a very friendly atmosphere, and the conference covered a wide range of topics, as can be seen in the summary above. A notable trend is the increasing number of different phage–host systems, witnessing a very active and growing community in France. Furthermore, the activity of our network extends beyond those yearly meetings, and several workshops took place including “Viromathon” organised by Annie Château and Marie–Agnès Petit, 18 October 2018 in Montpellier, “Phage ecology” organised by Mai Huong Chatain and Xavier Bellanger, 27 March 2019 in Lyon, “Genome sequencing and annotation” organised by Mireille Ansaldi and Marie–Agnès Petit, 8–9 October 2019 in Grenoble, and “Phage therapy applied to bone and joint infections” organised by Rémy Froissart and Tristan Ferry, 3 December 2019 in Lyon. During 2020, two workshops are already planned: “Phage Ecology” in Avignon, by Xavier Bellanger and Clara Tores–Barcelò, 27 March, and “Phage therapy” in Paris by Rémy Froissart, Aleandre Bleibtreu and Charlotte Brives, 25 June. More details on past and coming workshops are on our web site http://phages.fr/workshops/ Additionally, members of our community were present at a Specialised Scientific Committee of the French National Agency for Drug and Health Product Security to discuss “Phage therapy: feedback from experience and perspectives” (see talk of R. Delattre). Our 2020 conference is scheduled for 7–8 October, at the Pasteur Institute in Paris.

## Figures and Tables

**Table 1:** List of topics presented during the French Phage Network in Grenoble in 2019.

Poster Title	Authors. Presenters Underlined
Ecology and Evolution Session	
A molecular ecology approach using Stable Isotope Probing and metagenomics to study viruses of methanogens’ diversity	H. Ngo, M. Sotomski, M. Krupovic, F. Enault, O. Chapleur, Théodore B., A. Bize
Detection of an archaeal-specific viral family, previously thought to infect only hyperthermophiles, in human gut metaviromes	H. Ngo, C. Midoux, O. Rué, M. Mariadassou, V. Da Cunha, V. Loux, F. Enault, A. Bize
Genome analysis of *Pseudomonas* phage PPA2	ZE. Aynur, E. Oryasin, G. Basbülbül, B. Ertugrul, B. Bozdoğan
Proline and arginine metabolism at the interface of stationary phase physiology and bacteriophage infection in *Bacillus subtilis*	J. Dorling, A. Corral–Lugo, V. Cvirkaite-Krupovic, P. Tavares
**Phage Therapy and Biotechnology Session**	
DNA-free POETential, a synthetic biology project for the iGEM competition: Repurposing a DNA-less bacterium into an “RNA cell” with a little help from phages	A. Boudigou, C. Diaz, H. Herrmann, N. Moné, L. Maroc, M. Sabeti Azad, P. Bouloc, S. Bury–Moné, O. Rossier
Bacteriophages active against Meticillin Resistant *Staphylococci* isolated from bovine mastitis infections	Z. E. Aynur, G. Basbülbül, B. Bozdoğan
Strategy using phages to control *Staphylococcus aureus* responsible for bovine mastitis	M. H. Chatain
The use of interferometric microscopy to quantify viral particles in complex samples such as fecal filtrates	R. Sausset, M. Greffet, M–A. Petit, M. De Paepe
Assessing phage therapy against the plant pest *Xylella fastidiosa*	F. Clavijo, M–A. Jacques, M. Ansaldi
Study of the prophages of *Pseudomonas aeruginosa* strain PP001	M. Billaud, M–A. Petit, P. Champion–Arnaud
Optical Bacterial Susceptibility test by Surface Plasmon Resonance (SPR)	L. O’Connell
**Structure and Assembly Session**	
Detection of viral particles in bacterial cultures of *Xanthomonas campestris* pv. *campestris*	M. Kocanova, M. Baránek, A. Eichmeier
**Phage–Host Interaction Session**	
Exploring the mechanisms of host takeover by bacteriophage T5: role of the DNA-binding protein A2	M. Senarisoy, P. Cuniasse, S. Zinn–Justin, P. Boulanger
Investigation of A2 protein partners, an essential pre-early protein of bacteriophage T5	A. Djedid, N. Ginet, A. Battesti, M. Ansaldi
Filamentous phage translocation in *Escherichia coli* envelope does not require a functional TolQRA motor	P. Samire, B. Serrano, D. Duché, E. Lemarié, R. Lloubes, L. Houot
How bacteria and bacteriophage coexist in the mammalian gut?	M. Mansos Lourenco, L. Chaffringeon, Q. Lamy–Besnier, C. Eberl, B. Stacher, L. Debarbieux, L. De Sordi
Low efficiency of DNA mismatch repair system on lambda phage	M. De Paepe, J. Cornuault, M. Elez
ATP-dependent formation of Sak4 filaments: a first step towards the single strand annealing	O. Son, S. Baconnais, M–A. Petit, E. Le Cam, F. Lecointe
Role of H-NS in maintenance of Gifsy prophages lysogeny in *Salmonella enterica* ST4/74	J. A. Bulssico, M. Ansaldi, A. Wahl, A. Boulanger
Isolation and characterization of the bacteriophages infecting *Xanthomonas arboricola* pv. *juglandis*	I. Altin, K. Gasic, M. Krivokapic, E. Stefani
An early expressed *Pseudomonas* phage protein increases host susceptibility to lysis and antibiotics	M. De Jode, A. Chevallereau, M. Monot, E. Brambilla, G. Karimova, L. Debarbieux
Functional impact of relB-metK region and *clpP* carried by 12/111phiA prophage on GBS pathogenicity	A. Renard, M. Lacasse, S. Dos Santos Borges, N. van der Mee–Marquet
